# A protection motivation theory-guided telehealth coaching program for middle-aged adults with cardiometabolic risk: A feasibility trial

**DOI:** 10.1186/s12889-025-22238-w

**Published:** 2025-03-24

**Authors:** Zoe Ching-man Kwok, Hon-lon Tam, Benny Chung-ying Zee, Sally Wai-sze Lo, Fiona Wing-ki Tang, An Tao, Helen Yue-lai Chan

**Affiliations:** 1 The Nethersole School of Nursing, Faculty of Medicine, The Chinese University of Hong Kong, Shatin, New Territories, Hong Kong SAR China; 2 The Jockey Club School of Public Health and Primary Care, Faculty of Medicine, The Chinese University of Hong Kong, Hong Kong SAR, China; 3 Present Address: School of Nursing, University of Tasmania, Australia, Australia

**Keywords:** Cardiometabolic disease, Feasibility trial, Health coaching, Middle-aged adults, Primary prevention

## Abstract

**Background:**

Health coaching to address the escalated cardiometabolic risk in middle-aged adults in primary health care is underexplored. This study aimed to examine the feasibility and acceptability of a protection motivation theory-guided telehealth coaching program among middle-aged adults with cardiometabolic risks.

**Methods:**

This was a pretest–posttest study. The three-month intervention included four nurse-facilitated telehealth sessions tailored to individual cardiometabolic risks.

**Results:**

Thirty participants were recruited through social media and a community center. The eligibility and enrollment rates were 16.1% and 78.9%, respectively. Attrition at six months after enrollment was 33.3%, and intervention attendance was 82.5%. Most of the participants (76.7%) were satisfied with the program. Significant improvements were noted in the INTERHEART score for cardiometabolic risks, self-efficacy, anxiety, stress, and central obesity but not in health-promoting behaviors, depression, sleep quality, physical activity level, and physiological outcomes at six-month post-enrolment.

**Conclusion:**

A theory-based telehealth coaching was feasible and well-accepted among middle-aged adults, with potential in reducing cardiometabolic risks among middle-aged adults in primary care. This study revealed significant improvement in cardiometabolic risk, self-efficacy, anxiety, stress, and central obesity but changes for health-promoting behaviors, depression, sleep quality, physical activity level, and physiological outcomes were not noted. Progression to a definitive trial was supported with implication for future trials, including lowering the threshold of cardiometabolic risk to improve subject recruitment, change of assessment sessions to promote adherence to fasting instruction, and use of digital recording to facilitate health coaching process.

**Trial registration:**

: This trial was retrospectively registered on 05/07/2022 at ClinicalTrials.gov (identifier: NCT05444140).

## Background

Cardiometabolic diseases, which include cardiovascular disease, diabetes and chronic renal diseases, are the leading cause of global disability-adjusted life years in middle-aged populations [1–3]. Despite the serious sequelae, many cardiometabolic risk factors are related to lifestyle, such as low physical activity levels, unhealthy diet, stress, poor sleep and smoking, and are thus modifiable [4, 5]. These unhealthy lifestyles are common cardiometabolic risk factors in middle-aged populations, making them vulnerable to developing cardiometabolic disease [6]. The use of medication for controlling cardiometabolic risk factors, such as high blood pressure, blood glucose and lipid, were widely adopted in current practice [7]. Medicalization, the process of diagnosing and treating personal, behavioral and social issues as individual pathologies, facilitated individual and population health promotion and disease prevention over the decades [8]. However, over-medicalization which ignore the contextual factors in promoting healthy lifestyles should be avoided [9]. Therefore, we propose health coaching as an early preventive intervention to prevent pharmacological methods to control cardiometabolic risk.

Health coaching which aims to promote healthy lifestyles through enlightenment and empowerment has been widely adopted [10–12]. Studies have shown positive effects of health coaching on self-efficacy, mental health, weight management, and glycemic control among patients with chronic diseases [13–15]. However, our systematic review found that its application for preventing cardiometabolic risk in primary care has not been much studied [16]. The pooled results of health coaching revealed significant improvement in physical activity, but limited effects on the uptake of health-promoting behaviors and improvements in physiological and psychological health related to cardiometabolic risks. This may be explained by the low awareness and perceived cardiometabolic risk among middle-aged adults who are free from any formal diagnosis of cardiometabolic diseases [17]; therefore, existing health coaching programs underpinned by behavioral change models may not be persuasive to them.

Protection motivation theory (PMT), which emphasizes the cognitive appraisal processes, namely threat and coping appraisal, involved in mediating attitude and behavioral changes, has been used to predict health-related intentions, prevent diseases, and promote health [18–21]. Threat appraisal is the process of evaluating an individual’s perceived threat of a situation, such as their perceived vulnerability, perceived severity, fear arousal, and appraisal of rewards for not adopting a coping response. Coping appraisal is the process of evaluating an individual’s coping response to the perceived threat, such as perceived self-efficacy, perceived response-efficacy and perceived response-cost. Threat and coping appraisal together mediate the cognitive process and increase the protection motivation to perform the intended behaviors. Systematic reviews have reported that PMT-based interventions can effectively promote intention or behavioral changes to modify lifestyles, including exercise, diet, smoking cessation, alcohol abstinence, and adherence to treatment regimens or preventive strategies in primary care [18, 19]. PMT emphasizes the promotion of both threat and coping appraisal during the health coaching process to facilitate the motivation for health-promoting behavioral change. Hence, we developed a PMT-guided telehealth coaching program to increase the uptake of health-promoting behaviors for addressing cardiometabolic risks.

Telehealth coaching is defined as health coaching delivered via telehealth. Telehealth coaching significantly improved health outcomes, for example weight loss and glycemic control, as face-to-face health coaching did [22, 23]. Also, COVID-19 pandemic accelerated the use of telehealth coaching because people stayed at home and cannot access to face-to-face health care services [24]. Our systematic review revealed that the use of telehealth to deliver health coaching was found acceptable by middle-aged adults in primary care [16].

This trial aimed to examine the feasibility and acceptability of a protection motivation theory-guided telehealth coaching program among middle-aged adults with cardiometabolic risks. The findings will support the progression to definitive trials if proven feasible and acceptable.

## Methods

### Study aim

The aim of the study is to examine the feasibility and acceptability of a protection motivation theory-guided telehealth coaching program among middle-aged adults with cardiometabolic risks. Cardiometabolic risk is defined in this trial as the risk of developing cardiometabolic diseases. The non-laboratory INTERHEART risk score (IHRS) was used to screen the risk of developing cardiometabolic disease because it has been validated in international case-control studies for myocardial infarction and stroke risk [4, 5]. A strong association has also been found between the IHRS and cerebrovascular diseases detected in brain magnetic resonance imaging [25].

### Study design

A single-group pretest–posttest study was conducted in Hong Kong from April to November 2021. The trial was registered on 05/07/2022 at ClinicalTrials.gov (identifier: NCT05444140). The trial was reported according to the CONSORT extension to pilot and feasibility trials [26].

### Setting and population

The participants were recruited through social media, such as Facebook and Instagram, and the network of a community wellness center. The study was promoted through social media for subject recruitment to reach a wider group of potential participants. Interested people registered through an online form. A research assistant then contacted them through phone to explain the study and conduct eligibility screening. Eligible people were invited to the community wellness center to provide written consent and undergo baseline assessment.

People were recruited if they [1] were aged 40–64 years [2], at high risk of developing cardiometabolic diseases, as defined with a score of 16 or higher in the non-laboratory IHRS [3–5], could communicate in Cantonese, and [4] could give informed consent. In this trial, the non-laboratory IHRS was used to assess the risk of developing cardiovascular disease because it has been validated in international case-control studies for myocardial infarction and stroke risk [4, 5]. A strong association has also been found between the IHRS and cerebrovascular diseases detected in brain magnetic resonance imaging [25]. The total score ranges from 0 to 48, with scores of 0–9, 10–15, and ≥ 16 indicating low, moderate, and high risks, respectively. The score is calculated based on the weighted sum of nine risk factors, including age, sex, lifestyle, and psychosocial well-being, that are significantly related to cardiovascular diseases.

People were excluded if they were currently taking medication as prescribed to control hypertension, hyperlipidemia, or hyperglycemia; had been diagnosed with transient ischemic attack, stroke, myocardial infarction, atrial fibrillation, coronary heart disease, heart failure, or chronic renal failure; had been diagnosed with a terminal disease with a life expectancy of < 12 months; had eye or retinal disease that would affect the accuracy of retinal imaging, one of the outcomes; or were currently participating in another clinical trial or structured lifestyle modification program that would result in contamination bias.

### Sample size estimation

Based on the estimation according to the stepped rules of thumb for an intervention with a medium effect size and pooled effects of health coaching on physical activity in middle-aged adults [16, 27], the number of subjects required for a feasibility trial was 30.

### Health coaching program

A health coaching program was developed according to the findings from a systematic review on health coaching for cardiometabolic health among middle-aged adults studied [16] and key health coaching components identified in the literature [28, 29], underpinned by the PMT [18–21]. The health coaching program included four nurse-facilitated individual sessions delivered across three months, in weeks 1, 4, 8, and 12 (Table 1). Motivational interviewing (MI), a counseling approach used in health coaching for behavioral changes [30], was adopted [31, 32]. The four processes in MI, namely engaging, focusing, evoking, and planning, were applied during the health coaching process. In the first session, the health coach greets the participant to build a trusting relationship with the participant to engage the participant in the health coaching process. The health coach reviewed the cardiometabolic risk profile based on the baseline data with the participant and explored his/her understanding of cardiometabolic disease and risk factors. The visualization of the assessment results enabled understanding of the development of cardiometabolic diseases. The process promoted threat appraisal by increasing their awareness and perceived severity of his/her cardiometabolic risks. In the focusing process, the health coach guided the participant to focus on the direction for behavioral change and set the agenda of the health coaching session. The health coach evoked the participant’s intention to change by turning their ambivalence into motivation to change. Then, the health coach guided the participant to set individual goals and action plans through self-discovery which turned the intention to action into change. The health coach also guided the participant to explore facilitators and barriers to goal attainment and strategies for overcoming barriers. The process increased the participant’s coping appraisal by increasing their perceived self-efficacy, perceived response-efficacy and reducing their perceived response cost. The participant was given a booklet to record his/her goal and action plan for desired behavioral change. The participant was asked to document his/her progress of goal attainment using the booklet. In subsequent sessions, the health coach and participant reviewed the progress on goal attainment, and explored facilitators and barriers to goal attainment, and strategies to overcome barriers. The process promoted coping appraisal by increasing perceived self-efficacy and reducing perceived response costs.

**Table 1:** Outline of the Protection Motivation Theory-guided health coaching program

Health coaching process: (1) Preparation; (2) Greeting; (3) Setting the agenda; (4) Ask-tell-ask; (5) Action plan; (6) Closing the loop (in the last session: the health coach and participant summarize and conclude the health coaching process)		
**Source of information for the participant**: Participants’ booklet containing information on cardiometabolic disease, and to record the cardiometabolic risk profile, action plan, and goal attainment		
**PMT component**	**Purpose**	**Content**
***First session (1 h per session; week 1)***		
Threat appraisal	To increase the participant’s perceived vulnerability and perceived severity of developing cardiometabolic disease, fear arousal and moderate rewards in maladaptive coping.	The health coach and participant: 1. review the participant’s cardiometabolic risk profile and health-promoting behaviours. 2. explore the participant’s understanding of cardiometabolic disease and risk factors.
Coping appraisal	To increase the participant’s coping response to the appraised threat of developing cardiometabolic disease, perceived self-efficacy and perceived response-efficacy and reduce their perceived response cost.	The health coach helps the participant to: 3. set self-determined goals and an individualised action plan through self-discovery. 4. explore facilitators of, and barriers to goal attainment and strategies for overcoming barriers.
***Subsequent sessions (30 min per session; weeks 4***,*** 8***,*** and 12)***		
Threat appraisal	To increase the participant’s perceived vulnerability and perceived severity of developing cardiometabolic disease, fear arousal and moderate rewards in maladaptive coping.	1. The health coach and participant review the participant’s cardiometabolic risk profile and health-promoting behaviours, if necessary.
Coping appraisal	To increase the participant’s coping response to the appraised threat of developing cardiometabolic disease, perceived self-efficacy, and perceived response-efficacy and reduce their perceived response cost.	The health coach and participant: 2. review the progress and modify the goals and action plans as necessary through reflection. 3. review the facilitators of and barriers to goal attainment and the strategies to overcome barriers.

Due to social restrictions during the COVID-19 pandemic, the health coaching sessions were delivered through telehealth. The use of telehealth to deliver health coaching was found acceptable by middle-aged adults in primary care [16]. The proposed duration of the first session and subsequent sessions were 60 and 30 min, respectively, with reference to existing health coaching programs for middle-aged adults which demonstrated a trend of increased completion and significant improvement in behavioral change [16].

### Intervention fidelity

The intervention was delivered by the first author, a registered nurse with experience in primary care who had received health coach training. A health coaching protocol that outlined the health coaching process, techniques, and resources was developed to ensure consistency in intervention delivery across the participants. Four research assistants were trained to conduct outcome assessments independently, with satisfactory inter-rater reliability achieved (Cohen’s kappa coefficient = 1.0). Regular research team meetings were held to resolve any concerns regarding subject recruitment and data collection and thus ensure study quality.

### Feasibility

Feasibility was assessed using the eligibility rate (percentage of persons meeting the inclusion criteria out of those registered for eligibility screening), enrollment rate (percentage of participants who provided written consent out of the eligible persons), attendance rate (percentage of health coaching sessions attended out of the total sessions), attrition rate (percentage of participants who withdrew at three months and six months post-enrollment), and completion rate of outcome measures (percentage of participants who completed the outcome measure assessments out of all participants). Details of the outcome measures are described in a later section.

### Acceptability

Acceptability was measured using a self-developed 6-item questionnaire to assess the participants’ satisfaction with the health coaching program upon completion of the intervention. The participants rated the intervention in terms of the number and duration of health coaching sessions, duration of the whole program, perceived benefits of the program, and perceived motivation obtained for health behavioral change, and overall satisfaction using a 5-point Likert scale (0 = “strongly disagree” to 4 = “strongly agree”) through an online platform. They could also provide written responses to an open-ended question for any comment on the program.

### Measures

The following outcomes were measured at baseline and six months post-enrollment. Health-promoting behaviors were measured using four subscales of the Health-Promoting Lifestyle Profile II (HPLP II) pertaining to cardiometabolic risk, namely health responsibilities (9 items), nutrition (9 items), exercise (8 items), and stress (8 items) [33, 34]. Cardiometabolic risk was measured using the non-laboratory IHRS [4]. Self-efficacy in adopting health-promoting behaviors was measured using the adapted version of the Diabetes Mellitus Type II Self-Efficacy Scale [35]. Psychological distress was measured using the 21-item Depression Anxiety Stress Scale (DASS), which comprises three domains: depression (7 items), anxiety (7 items), and stress (7 items) [36–38]. The Pittsburg Sleep Quality Index (PSQI), which comprises 19 items under seven domains, was used to measure sleep quality over the past month [39, 40]. Physical activity level was measured using the International Physical Activity Questionnaire-Chinese (IPAQ-C), which comprises 11 items measuring physical activity at vigorous intensity and moderate intensity, walking, and sitting, over the past seven days [41].

Apart from self-reported outcomes, we also measured physiological parameters as objective biomarkers of cardiometabolic risks [42, 43]. These included blood pressure, body mass index (BMI), waist-to-hip ratio (WHR), point-of-care blood test after 8-hour fasting, and retinal imaging measured by a non-mydriatic fundus camera. BMI in the range of 23.0 − 24.9 and ≥ 25.0 were defined as overweight and obese, respectively [44]. WHR > 0.85 and > 0.90 for women and men, respectively, was defined as central obesity [44]. The point-of-care blood test for glucose, total cholesterol, and urate were widely used as cardiometabolic disease screening for the risk of developing diabetes, hyperlipidemia and renal diseases correspondingly [42, 43]. The retinal images were analyzed using Automatic Retinal Image Analysis (ARIA) to estimate stroke risk within minutes [45]. The ARIA-stroke algorithm has a sensitivity and specificity of 94.7% and 100%, respectively, to detect the risk of stroke based on a probability cut-off of 0.5 [45]. This technology supports user-friendly and accessible on-site stroke risk screening in primary care settings [46, 47].

### Sociodemographic data

Sociodemographic data, namely sex, age, marital status, work status, education level, and living arrangement, were collected at baseline.

### Progression to a definitive trial

The progression criteria were set, with reference to the current literature on health coaching program for middle-aged adults in primary care [16], to indicate the feasibility of progressing to a definitive trial. The thresholds of these criteria were set to achieve better than or equal to the lowest rate of eligibility, enrollment, attendance and attrition reported by similar studies. The progression criteria included:

1. Eligibility and enrollment rates of 20% and 60%, respectively;
2. An attendance rate of 80% for the intervention;
3. An attrition rate of < 40% at three months post-enrollment and six months post-enrollment; and.
4. An average satisfaction score of 3 out of 4.

### Statistical analysis

Descriptive statistics were used to present the participants’ characteristics, eligibility rate, enrollment rate, attendance rate, attrition rate, outcome measures completion rate, satisfaction survey results, and study outcomes. The baseline characteristics of the participants who completed the study and those who were lost to follow-up at six months post-enrollment were compared using the Mann–Whitney U test and Fisher’s exact test. The Wilcoxon signed-rank test or McNemar’s test was used to compare the differences in study outcomes between baseline and six months post-enrollment. Missing data was imputed with the mean of the corresponding variables. Data were analyzed using IBM SPSS version 28.0 (IBM Corp, Armonk, NY, USA). Significance was set at 0.05, and all the tests were two-tailed.

### Ethical considerations

Ethical approval was sought from the Research Ethics Committee (Chinese University of Hong Kong-NTEC Research Ethics Committee, CREC Ref. No. 2021.257). Written informed consent was sought from eligible and interested participants before the study. Participation was voluntary, and the participants could withdraw from the study at any time without reprisal.

## Results

### Participant flow

The eligibility rate was 16.1%. Of the 236 persons screened for eligibility, 198 were excluded because of having a non-laboratory IHRS < 16 (60.1%), having an eye disease that affected retinal imaging (5.1%), or currently taking medication to control their cardiometabolic disease (34.8%), giving an eligibility rate of 16.1%. Among the 38 eligible individuals, eight refused to participate because of their unavailability for the intervention, giving an enrollment rate of 78.9%. Therefore, progression criterion 1 was partially met. Five of the 30 participants withdrew from the study because of a busy schedule (*n* = 3), a plan for further medical follow-up (*n* = 1), or lack of interest in the intervention (*n* = 1). Additionally, two and three participants were lost to follow-up at three months and six months post-enrollment, respectively, giving attrition rates of 23.3% (7/30) at three months post-enrollment and 33.3% (10/30) at six months post-enrollment. Therefore, progression criterion 3 was met.

### Participants’ characteristics

The 30 participants had a mean age of 51.8 ± 6.3 years (range: 41–62 years) (Table 2). Most of them were men (73.3%), married (83.3%), employed (70%), living with others (93.3%), and had completed tertiary education (60.0%). A large proportion of the participants were overweight (36.7%) or obese (46.7%), and had central obesity (66.7%). Furthermore, 76.7% of the participants had no habit of self-monitoring blood pressure, and 96.7% had not undergone regular health checks.

**Table 2:** Sociodemographic and health characteristics of participants at baseline (*N* = 30)

	All (*N* = 30)	Completers (*n* = 20)	Non-completers (*n* = 10)	
	*n* (%)	*n* (%)	*n* (%)	*p*-value^a^
Age (years), mean ± SD (range)	51.8 ± 6.3 (41–62)	54.0 ± 5.6 (41–62)	47.7 ± 5.6 (41–57)	0.011
Sex				0.384^b^
Female	8 (26.7%)	4 (20.0%)	4 (40.0%)	
Male	22 (73.3%)	16 (80%)	6 (60%)	
Marital status				0.002^b^
Single/divorced/widowed	5 (16.7%)	0 (0%)	5 (50.0%)	
Married	25 (83.3%)	20 (100%)	5 (50.0%)	
Education level				1.000^b^
Secondary	2 (6.7%)	1 (5.0%)	1 (10.0%)	
High school	10 (33.3%)	7 (35.0%)	3 (30.0%)	
Tertiary	18 (60.0%)	12 (60.0%)	6 (60.0%)	
Work status				0.675^b^
Unemployed	9 (30.0%)	7 (35.0%)	2 (20.0%)	
Employed	21 (70.0%)	13 (65.0%)	8 (80.0%)	
Living alone				0.103^b^
No	28 (93.3%)	20 (100%)	8 (80.0%)	
Yes	2 (6.7%)	0 (0%)	2 (20.0%)	
Smoking habit				0.551^b^
Current smoker	5 (16.7%)	3 (15.0%)	2 (20.0%)	
Former smoker	7 (23.3%)	6 (30.0%)	1 (10.0%)	
Never	18 (60.0%)	11 (55.0%)	7 (70.0%)	
Habit of self-monitoring BP				1.0^b^
No	23 (76.7%)	15 (75.0%)	8 (80.0%)	
Yes	7 (23.3%)	5 (25.0%)	2 (20.0%)	
Habit of regular health check				1.0^b^
No	29 (96.7%)	19 (95.0%)	10 (100%)	
Yes	1 (3.3%)	1 (5.0%)	0 (0%)	
IHRS score, mean ± SD	19.0 ± 2.7	19.0 ± 2.8	19.1 ± 2.4	0.650
HPLP II score, mean ± SD				
Health responsibility	1.9 ± 0.4	1.8 ± 0.4	1.9 ± 0.4	0.948
Nutrition	2.2 ± 0.3	2.2 ± 0.3	2.2 ± 0.3	0.846
Exercise	1.7 ± 0.5	1.8 ± 0.6	1.6 ± 0.2	0.846
Stress	2.2 ± 0.5	2.3 ± 0.5	2.0 ± 0.3	0.155
Self-efficacy scale score, mean ± SD	3.4 ± 0.8	3.6 ± 0.8	2.8 ± 0.4	0.006
DASS score, mean ± SD				
Depression	8.2 ± 6.3	7.0 ± 4.9	10.6 ± 8.1	0.373
Anxiety	8.7 ± 5.3	6.8 ± 5.1	12.4 ± 3.2	0.005
Stress	13.7 ± 7.3	10.6 ± 5.3	20.0 ± 6.9	< 0.001
PSQI score, mean ± SD	8.0 ± 3.1	8.0 ± 3.2	8.0 ± 3.1	0.746
IPAQ-C category				1.000^b^
Inactive	13 (43.3%)	9 (45.0%)	4 (40.0%)	
Minimally active	8 (26.7%)	5 (25.0%)	3 (30.0%)	
Active	9 (30.0%)	6 (30.0%)	3 (30.0%)	
ARIA, mean ± SD	0.4 ± 0.1	0.4 ± 0.1	0.4 ± 0.1	0.588
BMI (kg/m^2^)	25.2 ± 3.5 (17.7–33.1)	25.6 ± 2.6 (21.1–33.1)	24.3 ± 4.8 (17.7–32.7)	0.448
BMI category				0.022^b^
Underweight (BMI < 18.5)	1 (3.3%)	0 (0%)	1 (10.0%)	
Normal (BMI 18.5–22.9)	4 (13.3%)	1 (5.0%)	3 (30.0%)	
Overweight (BMI 23.0–24.9)	11 (36.7%)	10 (50.0%)	1 (10.0%)	
Obese (BMI ≥ 25.0)	14 (46.7%)	9 (45.0%)	5 (50.0%)	
WHR	0.9 ± 0.1	0.9 ± 0.1	0.9 ± 0.1	0.779
Central obesity	20 (66.7%)	14 (70.0%)	6 (60.0%)	0.690^b^
SBP (mmHg)	127.7 ± 21.2	132.9 ± 15.0	117.3 ± 28.1	0.143
DBP (mmHg)	75.0 ± 14.6	74.9 ± 15.8	75.3 ± 12.6	0.880
Pulse rate (bests per minute)	71.6 ± 10.7	73.0 ± 10.5	68.8 ± 11.2	0.307
Point-of-care test				
Glucose (mmol/L)	5.8 ± 1.1	6.1 ± 1.2	5.1 ± 0.3	0.013
Total cholesterol (mmol/L)	5.3 ± 0.8	5.1 ± 0.6	5.7 ± 1.1	0.307
Urate (mmol/L)	0.4 ± 0.1	0.3 ± 0.1	0.4 ± 0.1	0.082

Notes: ^a^Mann–Whitney U test, unless specified; ^b^Fisher’s exact test; n (%), number (percentage); SD, standard deviation; ARIA, Automatic Retinal Image Analysis; BMI, body mass index; BP, Blood pressure; DASS, Depression Anxiety Stress Scale; DBP, diastolic blood pressure; HPLP II, Health-Promoting Lifestyle Profile II; IHRS, INTERHEART risk score; IPAQ-C, International Physical Activity Questionnaire–Chinese; PSQI, Pittsburgh Sleep Quality Index, SBP, systolic blood pressure; WHR, waist-to-hip ratio

Central obesity is defined as a waist-to-hip ratio > 0.85 and > 0.90 for women and men, respectively

### Comparison of completers and non-completers

The overall attendance rate for the health coaching sessions was 82.5%. Therefore, progression criterion 2 was met. The reasons for not attending sessions included sickness, having a busy schedule, withdrawal from the study, and being lost to follow-up. On average, the first session lasted 36 (range: 25–60) minutes and the subsequent sessions lasted 11 (range: 10–20) minutes. Compared with the 10 non-completers, the 20 participants who completed the study were likely to be older (*p* = 0.011), married (*p* = 0.002), overweight/obese (*p* = 0.022), had lower anxiety (*p* = 0.005) and stress levels (*p* < 0.001), higher self-efficacy (*p* = 0.006) and had higher blood glucose (*p* = 0.013) at baseline (Table 2).

### Acceptability

Twenty-three participants completed the satisfaction survey (Table 3). Most of them (76.7%) were satisfied/very satisfied with the health coaching program. The satisfaction scores ranged from 3.2 to 3.6 out of 4. Therefore, the progression criterion 4 was met. They generally agreed that the intervention’s dose was appropriate in terms of the number of sessions, length of each session, and duration of the program and that the program encouraged their health-promoting behaviors. In their qualitative comments to the open-ended question and reflection during the health coaching process, several participants appreciated the health coach for encouraging them in achieving their personalized goals throughout the program, particularly while having to accommodate their busy schedules.

**Table 3:** Satisfaction with the health coaching program

	Mean^a^ ± SD
1. The number of health coaching sessions was appropriate.	3.4 ± 0.6
2. The duration of each health coaching session was appropriate.	3.3 ± 0.6
3. The duration of the whole health coaching program was appropriate.	3.2 ± 0.8
4. The health coaching was helpful.	3.5 ± 0.6
5. The health coaching program motivated me to increase health-promoting behaviors.	3.5 ± 0.6
6. Overall, I am satisfied with this health coaching program.	3.6 ± 0.5

Notes: ^a^Possible range: 0–4; SD, standard deviation

### Completion rates of outcome measure

The outcome measure completion rates were 100% at baseline and 6 months post-enrollment after excluding those dropouts. The percentages of participants who followed the fasting instruction before the blood test were 26.7% and 36.7% at baseline and six months post-enrollment, respectively. The participants who attended the assessments in the afternoon mentioned the challenge of fasting since dinner the night before. Therefore, they either fasted for more than 20 h, which is much longer than the required 8 h before blood test or ate as usual and ignored the instruction.

### Preliminary effects

As shown in Table 4, significant changes were noted in the non-laboratory IHRS (*p* < 0.001), self-efficacy (*p* = 0.012), DASS–anxiety score (*p* < 0.001), DASS–stress score (*p* < 0.001), IPAQ-C category (*p* = 0.016), central obesity (*p* = 0.006) and point-of-care blood test result for urate (*p* = 0.024) at six-month post-enrollment when compared with baseline. Changes in the HPLP II score, DASS–depression score, PSQI score, ARIA reading, BMI, WHR, blood pressure, blood glucose, and total cholesterol were not significant.

**Table 4:** Outcomes at baseline and six-month post enrollment (*N* = 30)

Outcomes	Baseline^a^	Six-month post-enrollment	Changes	z	*p*-value^b^
	Mean ± SD	Mean ± SD			
HPLP II score					
Health responsibility	16.6 ± 3.7	17.1 ± 3.4	+ 0.50	1.00	0.318
Nutrition	20.0 ± 2.5	20.2 ± 3.2	+ 0.20	0.15	0.882
Exercise	13.5 ± 4.0	14.4 ± 2.9	+ 0.90	1.67	0.096
Stress	17.5 ± 3.6	18.2 ± 3.6	+ 0.70	1.48	0.140
Non-laboratory IHRS score	19.0 ± 2.7	14.0 ± 3.3	-5.00	-4.24	< 0.001
Self-efficacy scale score	3.4 ± 0.8	3.7 ± 0.6	+ 0.30	2.52	0.012
DASS score					
Depression	8.2 ± 6.3	6.5 ± 4.8	-1.70	-1.27	0.205
Anxiety	8.7 ± 5.3	5.1 ± 3.2	-3.60	-3.44	< 0.001
Stress	13.7 ± 7.3	8.7 ± 5.2	-5.00	-3.35	< 0.001
PSQI score	8.0 ± 3.1	8.7 ± 3.1	+ 0.70	0.99	0.324
IPAQ-C category, *n*(%)				-2.40	0.016
Inactive	13 (43.3%)	23 (76.7%)			
Minimally active	8 (26.7%)	3 (10.0%)			
Active	9 (30%)	4 (13.3%)			
ARIA	0.37 ± 0.10	0.34 ± 0.04	-0.03	-1.13	0.260
BMI (kg/m^2^)	25.2 ± 3.5	25.1 ± 1.9	-0.10	-0.40	0.688
BMI category, *n*(%)				-1.27	0.204
Underweight (BMI < 18.5)	1 (3.3%)	0 (0%)	-3.3%		
Normal (BMI 18.5–22.9)	4 (13.3%)	3 (10.0%)	-3.3%		
Overweight (BMI 23.0–24.9)	11 (36.7%)	10 (33.3%)	-3.6%		
Obese (BMI ≥ 25.0)	14 (46.7%)	17 (56.7%)	+ 10.0%		
WHR	0.91 ± 0.06	0.90 ± 0.05	-0.01	-1.41	0.159
Central obesity^#^, *n*(%)	20 (66.7%)	10 (33.3%)	-33.4%		0.006^c^
SBP (mmHg)	127.7 ± 21.2	125.7 ± 13.5	-2.00	-1.34	0.180
DBP (mmHg)	75.0 ± 14.6	74.2 ± 10.6	-0.80	-0.75	0.455
Pulse rate (beats per minute)	71.6 ± 10.7	73.4 ± 7.9	+ 1.80	1.56	0.119
Point-of-care blood test					
Glucose (mmol/L)	5.8 ± 1.1	5.9 ± 0.5	+ 0.10	0.98	0.325
Total cholesterol (mmol/L)	5.3 ± 0.8	5.6 ± 1.0	+ 0.30	1.24	0.213
Urate (mmol/L)	0.38 ± 0.08	0.41 ± 0.07	+ 0.03	2.27	0.024

Notes: ^a^ Values are the mean ± standard deviation or number (percentage); ^b^Wilcoxon signed-rank test, unless specified; ^c^McNemar’s test; ARIA, Automatic Retinal Image Analysis; BMI, body mass index; DASS, Depression Anxiety Stress Scale; DBP, diastolic blood pressure; HPLP II, Health-Promoting Lifestyle Profile II; IHRS, INTERHEART risk score; IPAQ-C, International Physical Activity Questionnaire–Chinese; PSQI, Pittsburgh Sleep Quality Index; SBP, systolic blood pressure; WHR, waist-to-hip ratio

^#^Central obesity is defined as a waist-to-hip ratio > 0.85 and > 0.90 for women and men, respectively

## Discussion

Our study attempts to advocate for health coaching to empower the middle-aged group to address cardiovascular risk in primary care. The findings suggested that the PMT-guided telehealth coaching program in primary care for middle-aged adults with cardiometabolic risks was feasible and well-accepted among the participants. Outcomes, including non-laboratory IHRS, self-efficacy, DASS–anxiety and stress score and central obesity were significantly improved at six months post-enrollment compared to baseline, but changes for HPLP II score, DASS–depression score, PSQI score, IPAQ-C score, ARIA reading, BMI, WHR, blood pressure, blood glucose, and total cholesterol were not noted.

The eligibility rate in our study was lower than those reported for existing health coaching programs designed for middle-aged adults in primary care [16]. The lower eligibility rate in this study might be because many people who met the inclusion criteria had already been put on medication to control cardiometabolic risk. This revealed the over-medicalization of cardiometabolic risk management although some risk factors are modified through lifestyle changes. Over-medicalization posed impediments to sustaining population health and health equality. Overdiagnosis, overprescription and unwanted side effects of medication, and ignorant to the social, economic and politic determinants of health are known shortcomings of over-medicalization [9]. Health coaching proactively promotes healthy lifestyles through enlightenment and empowerment in guiding the participants to explore facilitators and barriers to goal attainment through self-discovery. The process took into consideration the local context and participants’ own culture and beliefs in the health-promoting behavioral change for health promotion, instead of premature diagnosing and treating health risk with medicine for disease prevention. Therefore, health coaching for lifestyle change could be one of the strategies to overcome the over-medicalization of cardiometabolic risk management in primary care.

The percentage of male participants outgrew those reported in existing health coaching programs for middle-aged adults [16]. This study had addressed the research gap because gender bias was noted in existing health coaching for middle-aged adults in primary care which were predominantly female participants (99.1%) [16]. The finding of more men participated in this study was in concordance with existing clinical trials [48]. Mortality rate due to cardiovascular disease remained higher among middle-aged men than women [49]. Despite the gender differences, the proportion of women interested in the study was comparable to the proportion among those eligible for the study. Also, there was no significant difference in the completion rate between men and women. Among those withdrew from the study, female participants reported busy schedule due to caregiver role as the reason for withdrawal. Higher level of engagement in caregiving tasks among women might explain their lower completion rate in this study [50].

The attendance and attrition rates in our study were better than those reported for similar health coaching programs for middle-aged adults in primary care [11, 12]. The mean duration of the health coaching sessions was shorter than expected per protocol (first session and subsequent sessions were 60 and 30 min, respectively) because the participants had to manage some unforeseeable caregiving or work duties, requiring them to end the sessions earlier. This highlighted the importance of adopting a telehealth approach for health coaching conducted to cover the geographical constraints and allowed flexibility in scheduling to accommodate the busy lifestyles of middle-aged adults. The program’s acceptability was better than those reported for other health coaching programs for lifestyle changes [51, 52]. However, only a few participants used the booklet to record their progress in the action plan and goal attainment.

The participants who completed the study were more likely to be older, married, overweight/obese, had higher blood glucose and reported lower anxiety and stress levels and higher self-efficacy at baseline than the dropouts. Obesity and higher blood glucose are apparent cardiometabolic risk factors that probably increased the participants’ perceived health risk, thereby motivating them for behavioral changes. Studies have reported that being married and having lower anxiety and stress levels are associated with less maladaptive coping strategies, which may help to explain their higher likelihood of completing the study [53]. Furthermore, self-efficacy is fundamental to coping appraisal in PMT during the health coaching process [18–21]. The higher self-efficacy may have a mediating effect on the relationship between motivation and behavioral changes in people with cardiometabolic risks [54].

The outcome measure completion rate was better than the rates reported in similar studies (62.7–88.3%) [10–12]. The participants were generally able to understand and self-administer the questionnaires in this study. However, there was low adherence with the fasting instruction before the blood test. Some of the participants fasted for an unnecessarily long duration, while some did not follow the instruction and ate normally. Thus, the accuracy of the blood test results was affected.

The findings of significant improvement in cardiometabolic risk based on non-laboratory IHRS, stress and central obesity contributed valuable empirical evidence on the effects of health coaching in middle-aged adults in primary care because our systematic review found no previous studies reporting these outcomes [16]. The significant improvement in self-efficacy and anxiety was consistent with existing health coaching for middle-aged adults conducted in non-western countries [12] and those aimed to relieve anxiety [10], correspondingly. The inconclusive findings in physiological parameters in this study were consistent with the current evidence on the effects of health coaching for middle-aged adults in primary care [16]. The improvement in ARIA reading, BMI and blood pressure at six months post enrollment in this study, but not statistically significant, highlighted the potential of the program in improving biomarkers for cardiometabolic risk. The non-significant effects might be explained by the short-term evaluation of the intervention effects in this study because some statistically significant benefits of interventions can be detected only in long-term follow-up evaluation, for example cardiac event [55].

The proportion of participants classified as central obesity based on WHR was significantly reduced from two-thirds at baseline to one-third at six months post enrollment. There was also a reduction in the proportion of participants classified as overweight but not obese based on BMI. Both BMI and WHR are common anthropometric measurements adopted in health screening. BMI had been criticized for not taking lean mass and fat distribution into consideration for estimating severity of obesity related disease, despite its ease to use and inexpensive nature [56]. WHR, which measures the distribution of adiposity including visceral fat, is better predictor of obesity related disease and mortality [57]. Concurrent adiposity lost over waist area and/or muscle gained might explain the improvement in WHR, but no similar changes in BMI in this study. The discrepancy of these two results highlighted the use of reliable evaluation of body composition, such as bioelectrical impedance analysis [58].

### Study limitations

We acknowledge several limitations of this study. First, the enrollment and attrition rates may have been underestimated because of the lack of a control group, which otherwise might have increased refusal or attrition. Second, the participants’ feedback regarding feasibility and acceptability was based on quantitative questions. Therefore, the scope of the feasibility and acceptability measures was limited. Future definitive trials should explore the participants’ experience of health coaching with qualitative interview. Moreover, the point-of-care blood glucose levels should be interpreted cautiously due to the low rate of adherence to the instruction to fast for 8 h, which may have affected the accuracy of the results. Also, due to budget limitation, the less sensitive biomarker of point-of-care blood test for total cholesterol, was measured instead of the detailed examination of the lipid profile, including triglycerides and HDL cholesterol, in this study. A full-scale randomized controlled trial is crucial to confirm the effects of this PMT-guided telehealth coaching program on the health-promoting behaviors and cardiometabolic risk of middle-aged adults in primary care.

#### Implications for progression to future definitive trials

The high enrollment, attendance, and participant satisfaction rates and the low attrition rate in this trial indicate the feasibility of progressing to a definitive trial. Based on the experiences from this feasibility trial, we would like to propose the following changes in future definitive trial. First, we propose to lower the threshold of the non-laboratory IHRS score from 16 to 10, indicating a moderate risk of cardiovascular disease, for subject recruitment to address the low eligibility rate in this study [4]. In this study, the eligibility rate was low because many of them who had non-laboratory IHRS score 16 or above were already on medication management. Also, recruitment should be expanded to multiple centers in future definitive trial because recruitment through social media may limit access to people with higher digital literacy. Second, to promote adherence to the fasting instruction to ensure the accuracy of blood test results, the assessment sessions should be scheduled either in the morning or evening. Participants could fast after dinner the previous night or after breakfast/brunch for a blood test in the evening. Third, given the low utilization of the booklet by the participants, an alternative, such as the use of digital recordings via mobile application, is recommended to record their goals and action plans to improve their engagement [59].

## Conclusion

This feasibility trial showed that the PMT-guided telehealth coaching program was feasible and well-accepted among middle-aged adults. This trial reported the potential effects of the program in improving the cardiometabolic risk, self-efficacy, anxiety, stress levels and central obesity. However, non-significant changes in health-promoting behaviors, depression levels and physiological parameters including ARIA, anthropometric measures, blood pressure, blood glucose and total cholesterol were found. A fully powered robust clinical trial is warranted to test the effects of this program on the primary prevention of cardiometabolic disease in middle-aged adults. Implications for future trials include lowering the threshold of cardiometabolic risk for subject recruitment, scheduling the assessment sessions in the morning or evening to promote adherence to fasting instructions, and use of digital recording to facilitate health coaching process.

## Data Availability

The datasets used and/or analysed during the current study are available from the corresponding author on reasonable request.

## Declarations

### Clinical trial registration

This trial was retrospectively registered on 05/07/2022 at ClinicalTrials.gov (identifier: NCT05444140). https://clinicaltrials.gov/ct2/show/NCT05444140?term=NCT05444140%26;draw=2%26;rank=1.

## Abbreviations

PMT: Protection Motivation Theory
IHRS: INTERHEART risk score
HPLP II: Health-Promoting Lifestyle Profile II
ARIA: Automatic Retinal Analysis
DASS: Depression Anxiety Stress Scale
PSQI: Pittsburg Sleep Quality Index
IPAQ-C: International Physical Activity Questionnaire-Chinese
BMI: Body mass index
WHR: Waist-to-hip ratio
